# Localized recovery of complex networks against failure

**DOI:** 10.1038/srep30521

**Published:** 2016-07-26

**Authors:** Yilun Shang

**Affiliations:** 1 Department of Mathematics, Tongji University, 200092 Shanghai, China

## Abstract

Resilience of complex networks to failure has been an important issue in network research for decades, and recent studies have begun to focus on the inverse recovery of network functionality through strategically healing missing nodes or edges. However, the effect of network recovery is far from fully understood, and a general theory is still missing. Here we propose and study a general model of localized recovery, where a group of neighboring nodes are restored in an invasive way from a seed node. We develop a theoretical framework to compare the effect of random recovery (RR) and localized recovery (LR) in complex networks including Erdős-Rényi networks, random regular networks, and scale-free networks. We find detailed phase diagrams for the subnetwork of occupied nodes and the “complement network” of failed nodes under RR and LR. By identifying the two competitive forces behind LR, we present an analytical and numerical approach to guide us in choosing the appropriate recovery strategy and provide estimation on its effect by using the degree distribution of the original network as the only input. Our work therefore provides insight for quantitatively understanding recovery process and its implications in infrastructure protection in various complex systems.

Interconnection between network nodes has important consequences for stability and function of many complex networked systems ranging from Internet and social networks to electric power grids and transportation systems, where nodes are prone to random failure or malicious attack^1^,^2^,^3^,^4^. Through these interconnections, failure of individual components may crucially affect the performance of the whole system. The Internet with a scale-free connectivity distribution, for example, is highly vulnerable to attacks targeted at hubs but extremely robust against random errors^1^,^5^. Due to practical importance for applications, robustness of complex networks to failures remains a topic of central interest in the network science literature^6^,^7^,^8^,^9^,^10^,^11^,^12^.

While most efforts have been focused on probing the influence of network topology on robustness, failure mechanism of complex networks is very recently incorporated into the picture. It is revealed that an entire class of real-world networks, including human brains, cancer networks, polymers, financial systems, and botnets are capable of spontaneously recovery (or self-healing) after their collapse by restoring the failed nodes or edges^13^,^14^. The work^13^ describes a phase-flipping phenomenon between “active” and “inactive” collective modes in a time-dependent Erdős-Rényi networks using a threshold rule, where each node becomes inactive (or removed) if it has less than a certain fraction of active neighbors and it then can be recovered after a fixed time period. An extension to dynamic scale-free networks is presented in ref. 15. Self-healing capability of nodes using a related threshold rule has been dealt within percolation theory^14^. Ref. 16 studies the targeted recovery algorithm in which the hub nodes in interdependent networks are iteratively recovered. A recovery strategy that repairs failed nodes and edges randomly in the boundary of the largest connected component is introduced in ref. 17, where the critical probability of recovery that halts the cascade is identified for interdependent networks.

Different from recovering missing nodes or edges to restore functionality, another approach to improving network robustness consists in adding new edges or smartly modifying the network topology^18^,^19^,^20^,^21^,^22^. For instance, optimal recovery algorithms of adding minimum links so that every node can reach any other node in the resulting network is studied in ref. 18. However, as pointed out in refs 14,23, the possibility to create new links is normally not available in many real networks such as infrastructure networks, where physical links are fixed in advance and creating new ones incurs time and investments.

In this paper, we tackle the robustness of complex networks by considering *localized recovery* (LR) strategy, and comparing it with *random recovery* (RR) strategy. The localized recovery consists of a healing process of one node, then its neighbors, and then their neighbors, etc. (see Fig. 1). It restores only failed nodes and edges without assuming any particular healing mechanisms, e.g., the threshold rules^13^,^14^,^15^, hence addressing the shortcomings associated with both approaches. Localized recovery strategy is widely employed in the real world because (a) it is usually easier and more effective to repair a node in the neighborhood than one far apart as functional nodes become connected after recovery and (b) it is sometimes the only way to repair when an external monitor/controller is not available to guide the recovery or limited resources fail to maintain simultaneous recovery in multiple unrelated areas.

The analytical framework developed here for studying localized recovery of complex networks allows us to examine some percolation properties such as the critical recovery probability *r*_*c*_, marking the threshold of existence/non-existence of giant component in the network of fail nodes, and the fraction *P*_∞_ of failed nodes in the giant component of the network of failed nodes after recovery. Let *r*_0_ be a second critical recovery probability signifying the threshold of the network of occupied (i.e., functional or recovered) nodes, at which a giant component of occupied nodes first forms. As we will see below, *r*_*c*_ is distinct from the threshold *r*_0_, which is essentially trivial under LR. We find detailed phase diagrams under RR and LR. By identifying the two competitive forces that influence the effect of localized recovery strategy, we develop an analytical tool that can guide us in choosing the appropriate recovery strategy and provide estimation on its effect by using the degree distribution of the original network as the only input. Real networks with degree-degree correlation are also investigated. Our theoretical calculations are confirmed by extensive numerical simulations.

We mention that the localized recovery strategy hinges on the interconnections in a network, especially the topology of its shells^24^, which is defined as the set of nodes that are at some distance from a randomly chosen root node. Shell structure has been explored recently by some researchers to collapse an otherwise connected network^25^,^26^,^27^,^28^.

## Results

### Description of the model

The interconnection topology of the random network considered here is characterized by an arbitrary degree distribution *P*(*k*), which is the probability that a randomly chosen node has degree *k*. Starting from a randomly selected node (root), all nodes are listed in ascending order of their distances from the root. The set of nodes that are at distance *l* from the root is referred to as shell *l*^24^. Nodes that are at the same distance from the root are deployed randomly in the same shell.

**Figure 1:**
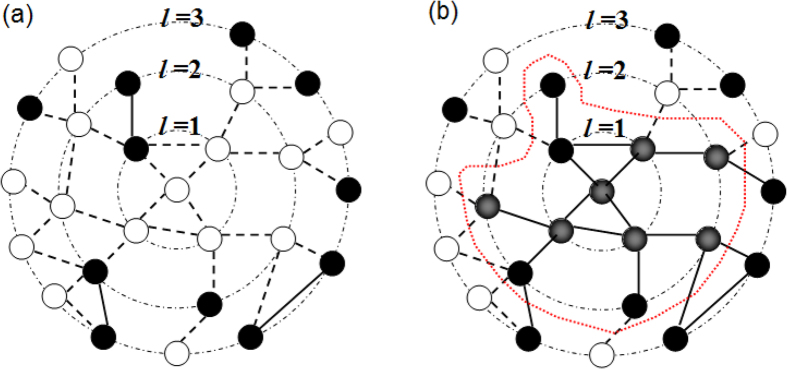
Schematic illustration of the localized recovery process. (**a**) A network suffers from random failures, where each node remains functional with probability *q* (black means functional nodes, white failed nodes); the root node is failed in this case. (**b**) A fraction *r* of the failed nodes are recovered starting from the root, its nearest neighbors, next nearest neighbors, and so forth (gradient black means recovered nodes); edges connecting two occupied (i.e., functional or recovered) nodes become active (solid lines) again. The recovery area is indicated by a red contour.

We assume that the network suffers from random failures after which a fraction *q* of nodes is functional (see Fig. 1(a)). Our localized recovery process is initiated by first checking the root node, then the nodes in the first shell, and so forth in the ascending order of their distance from the root according to the following rules:

1. Each failed node under check is recovered, and the edge connecting two occupied (i.e., functional or recovered) nodes become active (i.e., present) automatically.
2. Nodes in the same shell are checked randomly and we begin checking nodes in shell *l* + 1 only after all nodes in shell *l* are checked.

We continue the recovery process until a fraction *r* of failed nodes are recovered. Hence, a recovery area appears around the root node, and the remaining 1 − *r* fraction of failed nodes are at larger distance from the root (see Fig. 1(b)).

The key observation here is that the critical recovery fraction *r*_0_(LR) (Here, *r*_0_(LR) means *r*_0_ under LR; similar notations will be used for *r*_*c*_, *P*_∞_, and RR, etc.) signifying the emergence of a giant component of occupied nodes is equal to zero for any *q* ∈ [0, 1] when the original network is connected, i.e., having a single component. This observation motivates our interest in a closely related critical recovery fraction *r*_*c*_(LR), pointing to the threshold of a giant component in the network of failed nodes after recovery. This sort of study has important practical relevance since it informs us when the failures (such as epidemics in a population or virus in cyber spaces) can be controlled/confined to local areas, as well as a prediction of full system restoration.

We also consider the random recovery strategy as a comparison, where each initially failed node is recovered with probability *r* independently of each other, and edges connecting two occupied (i.e., functional or recovered) nodes are present automatically. The random recovery process can be easily accommodated by the classical node percolation theory^29^ with occupation probability *q* + *r* − *qr*. Following refs 29,30, the probability generating functions of node degree and excess node degree are defined as 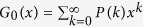 and 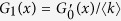, respectively, where 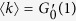 is the average degree of the original network. We find that the critical recovery probability is (see Supplementary Note A)


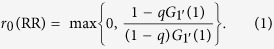


Hence, when 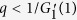, the initial random failure collapses the network, and the RR process plays a role in integrating the network.

### Network of failed nodes under RR

Recall that the generating function for the degree distribution of the original network is 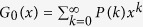. Based upon the concept of induced subgraph on the set complement in graph theory, the induced network of failed nodes under RR can be readily viewed as the result of independent node percolation^29^ with “occupation” probability (1 − *q*)(1 − *r*). Recall that (1 − *q*)(1 − *r*) is the fraction of failed nodes after recovery, and hence it is the probability that a node is in the complement network (composed of failed nodes). Under LR, for example, the complement network is the induced subnetwork of white nodes depicted in Fig. 1(b).

If we focus on the complement network, following ref. 29, the size distribution of the clusters that can be reached following a randomly chosen edge is generated in a self-consistent equation


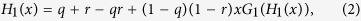


where 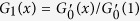. Here, the first term *q* + *r* − *qr* represents the probability that a node is occupied, namely, not in the complement network. The second term (1 − *q*)(1 − *r*)*xG*_1_(*H*_1_(*x*)) addresses the situation where the edge lead to a failed node with *k* other edges leading out of it, distributed according to the generating function (1 − *q*)(1 − *r*)*G*_1_(*x*). Analogously, the size distribution of the cluster to which a randomly chosen node belongs is generated by


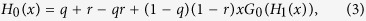


where the second term (1 − *q*)(1 − *r*)*xG*_0_(*H*_1_(*x*)) addresses the situation where the chosen node is failed (hence included in the complement network), and it has *k* other edges leading out of it, distributed according to the generating function (1 − *q*)(1 − *r*)*G*_0_(*x*). Then the critical recovery probability *r*_*c*_, at which a giant component of the network of failed nodes emerges, is determined by


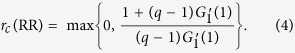


When the initial random failure is not severe, i.e., 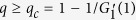, the network of failed nodes remains small components even without recovery (since *r*_*c*_ = 0). In general, a giant component of failed nodes first forms when *r* < *r*_*c*_.

**Figure 2:**
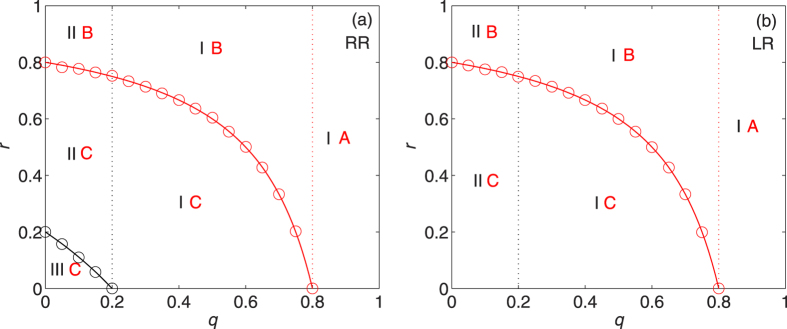
Phase diagrams in the *q*-*r* plane for ER networks with *λ* = 5 under (a) RR and (b) LR strategies. The meanings of the combined phases are described in Tab. 2. Solid lines are analytical results, from (1) for *r*_0_ (black line) and (6) for *r*_*c*_ (red lines). Data points (black and red circles) correspond to the simulation results averaged over 30 random graphs with 20 independent realizations for each.

Comparing the two thresholds (1) and (4), we observe interestingly that the critical point 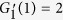 separates a regime of *r*_0_(RR) > *r*_*c*_(RR) and that of *r*_0_(RR) < *r*_*c*_(RR). The quantity 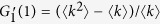 is called the branching factor of the original network, specifying the average number of outgoing links of the underlying branching process^2^,^30^. In a sparse original network with 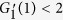, we have *r*_0_(RR) > *r*_*c*_(RR). This can be intuitively explained as follows. With the increase of the recovery fraction *r*, the giant component in the network of failed nodes first collapses and then a giant component in the network of occupied nodes forms. In other words, there is a coexistence phase where both networks have only small components. This is due to the sparseness of the network. While in a dense original network with 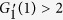, we have *r*_0_(RR) < *r*_*c*_(RR) indicating a coexistence of giant components in both subnetworks of occupied nodes and failed nodes. (See Fig. 2(a) and Supplementary Note C for the evolution of phase diagrams).

The fraction *S* of the giant component in the network of failed nodes satisfies


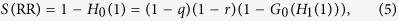


where *H*_1_(1) satisfies *H*_1_(1) = (*q* + *r* − *qr*) + (1 − *q*)(1 − *r*)*G*_1_(*H*_1_(1)). We denote by *P*_∞_ the relative size of the giant component as a fraction of the original network. Clearly, *P*_∞_(RR) = *S*(RR).

In what follows we instantiate the above formula (4) and (5) in the special cases of ER and REG networks (see Methods section for definitions). For ER networks, *G*_0_(*x*) = *G*_1_(*x*) = *e*^*λ*(*x*−1)^, and the critical recovery probability is


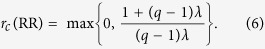


The relative size of the giant component satisfies


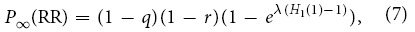


where *H*_1_(1) satisfies 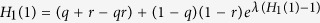. For REG networks, 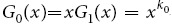, and the critical recovery probability is


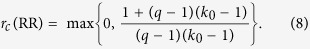


Similarly, the relative size of the giant component satisfies


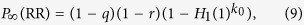


where *H*_1_(1) satisfies 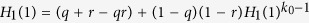.

### Network of failed nodes under LR

As in the RR scenario above, the critical recovery probability *r*_*c*_(LR) = 0 (i.e., no recovery is virtually needed to maintain the functionality of the original network) when the initial random damage is below a threshold, namely, 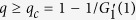. We find that *r*_*c*_(LR) satisfies (see Supplementary Note B)


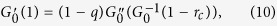


when 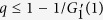. Recall that *r*_0_(LR) ≡ 0, and hence the inequality *r*_0_(LR) ≤ *r*_*c*_(LR) always holds, which is in sharp contrast to the RR case. Clearly, whenever the branching factor 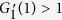, there is a co-existence phase where both subnetworks of occupied nodes and failed nodes have giant components (see the figures below for ER, REG and SF networks, and Methods section for the definition of these networks).

Furthermore, we find that the relative size of the giant component as a faction of the original network is (see Supplementary Note B)


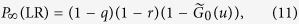


where *u* satisfies 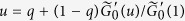, and


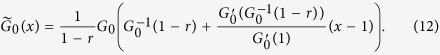


In what follows we derive explicit analytical expressions for the formula (10) and (11) in the special cases of ER and REG networks. For ER networks, 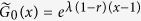 using (12). The critical recovery probability *r*_*c*_(LR) = *r*_*c*_(RR) which is given by (6). The relative size of the giant component satisfies


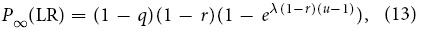


where *u* satisfies *u* = *q* + (1 − *q*)*e*^*λ*(1−*r*)(*u*−1)^. For REG networks, 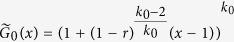, and the critical recovery probability is


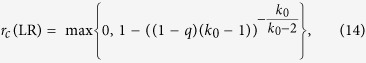


for *k*_0_ > 2 and *r*_*c*_(LR) = 0 for *k*_0_ = 2. The relative size of the giant component satisfies


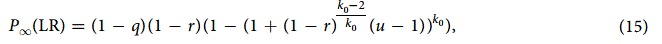



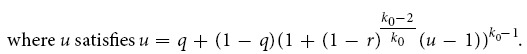
.

### ER networks under RR and LR

Figure 2 shows the (combined) phase diagrams for ER networks with average degree 〈*k*〉 = 5 under RR and LR (see also Table 1). The lines of the critical recovery probabilities *r*_0_ and *r*_*c*_, in good agreement with the simulation results, separate the regimes of (non)existence of giant component in the network of occupied nodes and in the network of failed nodes, respectively. As expected from the theoretical prediction, under RR there is a coexistence phase (i.e., the broken ring-shaped part between the red and black lines in Fig. 2(a)) where both networks have giant components since 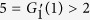. This coexistence phase disappears when 〈*k*〉 = 2 and a different coexistence phase where both networks are absent of giant components emerges when 〈*k*〉 < 2 (see Supplementary Note C).

Comparing Fig. 2(b) with Fig. 2(a), we observe that the combined phase IIIC only exists under RR. This means that the network of occupied nodes possesses a giant component as long as *r* 0 under LR, which constitutes a unique feature of LR. On the other hand, the two curves *r*_*c*_(RR) and *r*_*c*_(LR) coincide, which indicates that RR and LR strategies have the same healing capability (in terms of *r*_*c*_) for ER networks.

**Figure 3:**
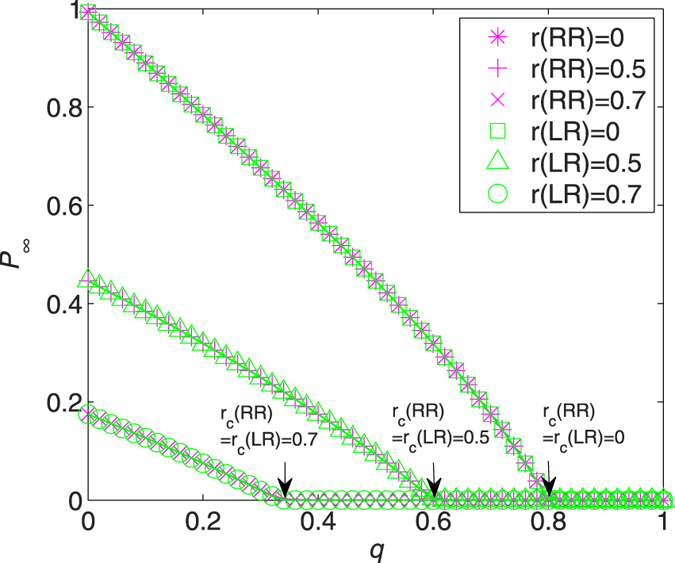
Relative sizes of giant component in the network of failed nodes, *P*_∞_, as a function of *q* for ER networks with *λ* = 5 and *r*(RR) = 0 (stars), *r*(RR) = 0.5 (pluses), *r*(RR) = 0.7 (crosses), *r*(LR) = 0 (squares), *r*(LR) = 0.5 (triangles), and *r*(LR) = 0.7 (circles). Solid lines are analytical results, from (7) for RR (magnet lines) and (13) for LR (green lines). Symbols correspond to the simulation results averaged over 30 random graphs with 20 independent realizations for each. The critical recovery fractions are indicated by arrows.

The effect of RR and LR is better appreciated when turning to the results reported in Fig. 3. Several interesting comments can be drawn. First, in all cases the simulation results confirm the theoretical predictions. The equalities *r*_*c*_(RR) = *r*_*c*_(LR) (arrows in Fig. 3) hold, and these critical points agree with those displayed in Fig. 2. For example, recall that *q*_*c*_ is the critical value at which *P*_∞_ vanishes, and we have *q*_*c*_ = 0.8 for *r*_*c*_ = 0 and *q*_*c*_ ≈ 0.6 for *r*_*c*_ = 0.5. Second, the fraction of giant component *P*_∞_ in the network of failed nodes displays identical behavior under RR and LR for *r* = 0, 0.5, and 0.7. In fact, such equivalence holds for all *q*, *r* ∈ [0, 1] (see Supplementary Note D for a direct proof), meaning that RR and LR has exactly the same effect in containing the damage of an ER network. This can be interpreted as a detailed balance between two competitive forces: (i) the nature of the LR that nodes within the recovery area are connected to each other while only exterior nodes linked to the surface of the recovery area contribute to the network of occupied nodes (namely, the “recovering resistance”), and (ii) the level of degree heterogeneity, where a larger-degree node is more likely to be recovered under LR, leading even more likely to an occupied giant component (namely, the “recovering impetus”).

It is worth mentioning that a balance phenomenon for ER networks under random and localized attacks has been observed recently by Shao *et al*.^26^. Their results correspond to the coincidence of *P*_∞_(RR) and *P*_∞_(LR) at the point *q* = 0 in Fig. 3. Note that if all nodes are removed initially (i.e., *q* = 0), the giant component in the network of failed nodes after random (or resp., localized) recovery considered here is exactly that in the network of remaining nodes under random (or resp., localized) attack in an original intact network.

### REG networks under RR and LR

**Figure 4:**
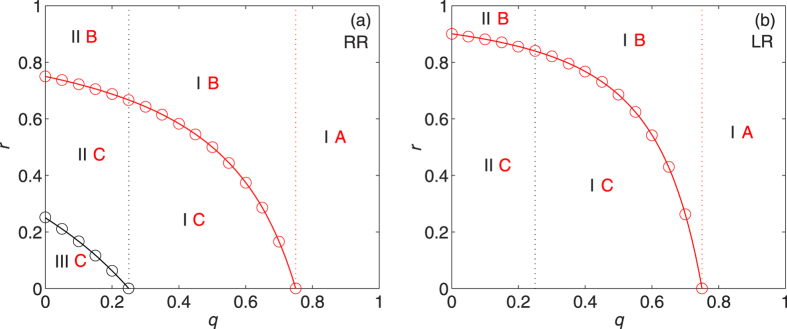
Phase diagrams in the *q*-*r* plane for REG networks with *k*_0_ = 5 under (a) RR and (b) LR strategies. The meanings of the combined phases are described in Table 1. Solid lines are analytical results, from (1) for *r*_0_ (black line) and (8), (14) for *r*_*c*_ (red lines). Data points (black and red circles) correspond to the simulation results averaged over 30 random graphs with 20 independent realizations for each.

Figure 4 shows the (combined) phase diagrams for REG networks with constant node degree *k*_0_ = 5 under RR and LR. The lines of the critical recovery probabilities *r*_0_ and *r*_*c*_ agree very well with the simulation results. As anticipated, the coexistence phase (phases IC and IIC) of giant components in both networks appears under RR since 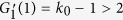 (see Fig. 4(a)). Fig. 4(b) again reflects the fact that *r*_0_(LR) ≡ 0. Comparing Fig. 4(a) with Fig. 4(b), we find that *r*_*c*_(RR) < *r*_*c*_(LR) for all *q* < *q*_*c*_ = 0.75. This means that LR is always less powerful than RR, namely, more nodes have to be recovered under LR in order to confine the damage to small/scattered areas.

**Figure 5:**
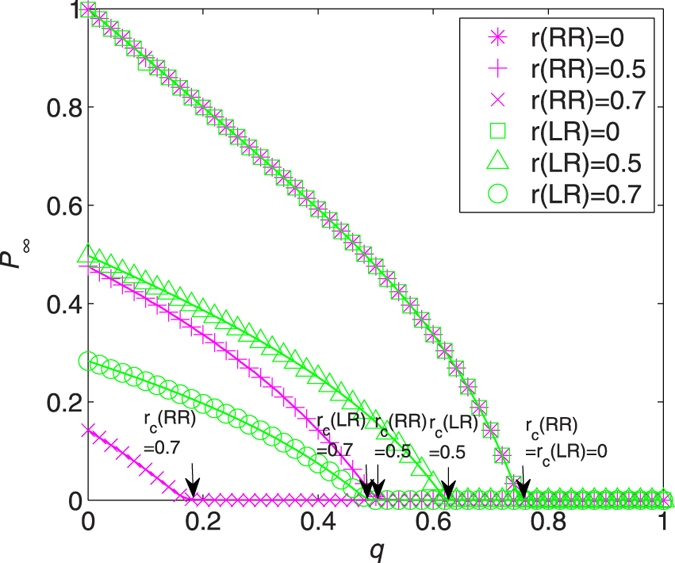
Relative sizes of giant component in the network of failed nodes, *P*_∞_, as a function of *q* for REG networks with *k*_0_ = 5 and *r*(RR) = 0 (stars), *r*(RR) = 0.5 (pluses), *r*(RR) = 0.7 (crosses), *r*(LR) = 0 (squares), *r*(LR) = 0.5 (triangles), and *r*(LR) = 0.7 (circles). Solid lines are analytical results, from (9) for RR (magnet lines) and (15) for LR (green lines). Symbols correspond to the simulation results averaged over 30 random graphs with 20 independent realizations for each. The critical recovery fractions are indicated by arrows.

The insight that RR is more effective for healing an REG network is better fathomed when it comes to Fig. 5. Similarly as in ER networks, the critical points of percolation, *r*_*c*_(RR) and *r*_*c*_(LR) (indicated by arrows), agree with those displayed in Fig. 4. In addition to these discrete points, the whole curve of *P*_∞_ follows the same pattern that *P*_∞_(RR) < *P*_∞_(LR) for *r* > 0. This can be easily understood as there is no “recovering impetus” (the degrees are all the same in REG networks); the “recovering resistance” renders LR inferior for healing REG networks after any degree of damage.

### SF networks under RR and LR

**Figure 6:**
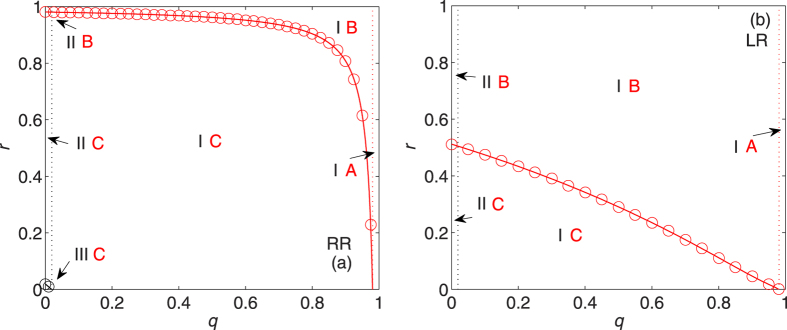
Phase diagrams in the *q*-*r* plane for SF networks with *γ* = 2.4, *k*_min_ = 2, and 〈*k*〉 = 5.08 under (a) RR and (b) LR strategies. The meanings of the combined phases are described in Tab. 2. Solid lines are analytical results, from (1) for *r*_0_ (black line) and (4), (10) for *r*_*c*_ (red lines). Data points (black and red circles) correspond to the simulation results averaged over 30 random graphs with 20 independent realizations for each.

In Fig. 6 we plot the (combined) phase diagrams for SF networks with *γ* = 2.4, *k*_min_ = 2, and 〈*k*〉 = 5.08 under RR and LR. The simulations for the critical recovery probabilities *r*_0_ and *r*_*c*_ are consistent with theoretical results. Similarly as in ER and REG networks, the coexistence phase (phases IC and IIC) of giant components in both networks appears under RR (see Fig. 6(a)) since 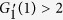 (which is actually more than 50 here).

Remarkably, the phases IA, IIB, IIC, IIIC under RR (Fig. 6(a)) and the phases IA, IIB, IIC under LR (Fig. 6(b)) are very small yet not negligible. This phenomenon is known to be the finite-size effect^2^,^31^. For an infinite SF network with power-law distribution 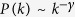, the ratio 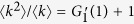 diverges when 1 ≤ *γ* < 3. So, in an infinite SF network, we have 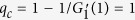, meaning that for any *q* > 0 the network of failed nodes always has a giant component (hence is extremely robust^2^,^5^) even without recovery (i.e., *r* = 0). When the recovery process is taken into consideration, the red curve *r*_*c*_ divides the whole phase plane into two regions: IB, where the network of failed nodes has no giant component and IC, where it has a giant component. Since *r*_*c*_(LR) < *r*_*c*_(RR) for all *q*, we conclude that LR is more powerful than RR for healing SF networks, opposite to the best practice for healing REG networks.

**Figure 7:**
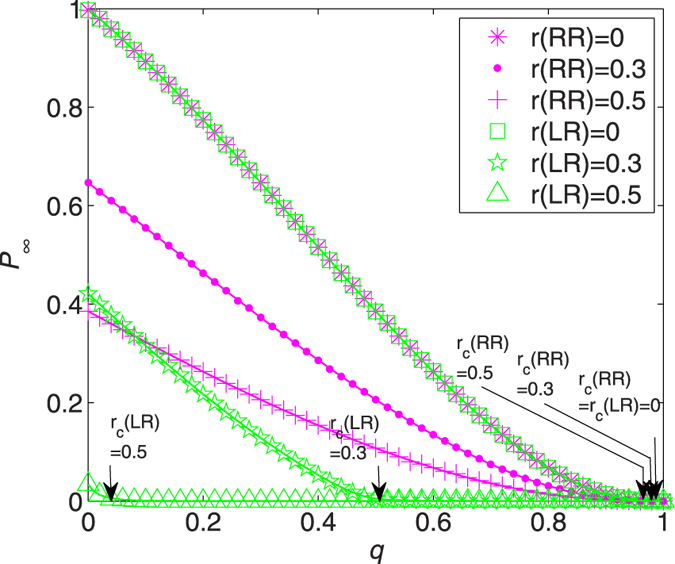
Relative sizes of giant component in the network of failed nodes, *P*_∞_, as a function of *q* for SF networks with *γ* = 2.4, *k*_min_ = 2, 〈*k*〉 = 5.08 and *r*(RR) = 0 (stars), *r*(RR) = 0.3 (dots), *r*(RR) = 0.5 (pluses), *r*(LR) = 0 (squares), *r*(LR) = 0.3 (pentagrams), and *r*(LR) = 0.5 (triangles). Solid lines are analytical results, from (5) for RR (magnet lines) and (11) for LR (green lines). Symbols correspond to the simulation results averaged over 30 random graphs with 20 independent realizations for each. The critical recovery fractions are indicated by arrows.

In addition to *r*_*c*_, the entire curve of *P*_∞_ follows the same pattern that *P*_∞_(LR) < *P*_∞_(RR) for *r* > 0 (see Fig. 7), meaning that the superiority of LR consists not only in the threshold but also in the size of giant component. Clearly, the level of degree heterogeneity (i.e., the “recovering impetus”) is dominant in SF networks: recovered hub nodes accelerate the recovery of the original network (hence rapidly collapse the network of failed nodes).

When the scaling exponent *γ* in an SF network is getting larger, the network becomes less heterogeneous. A phenomenon similar to that in REG networks, e.g., *r*_*c*_(LR) > *r*_*c*_(RR), is expected for a large *γ* (see Supplementary Note E). Then it is possible to determine a critical exponent *γ*_*c*_ = *γ*_*c*_(*q*) so that *r*_*c*_(RR) = *r*_*c*_(LR) at *q*. Indeed, if a given network has a power-law degree distribution with exponent *γ*, we can identify *γ*_*c*_(*q*) for any *q* ∈ [0, 1] by using Eqs (4),(10) and parameters in *P*(*k*) such as *k*_min_ and *k*_max_. Likewise, we can identify another critical exponent *γ*_∞_ = *γ*_∞_(*q*, *r*) so that *P*_∞_(RR) = *P*_∞_(LR) at any desired values of *q* and *r*. By comparing *γ*, *γ*_*c*_(*q*), and *γ*_∞_(*q*, *r*), we are able to determine the best recovery strategy (RR or LR) and acquire a good estimation on its effect under any level of initial failure as well as recovery in the network by using the degree distribution of the original network as the only input. Applications to some real-world networks with different sizes can be found in Supplementary Note F.

## Discussion

We have proposed and studied the localized recovery strategy for complex networks under random failures. Based on the generating function formalism, we tackle analytically the percolation properties associated with both the network of occupied nodes and the “complement network” of failed nodes. Detailed phase diagrams under RR and LR are obtained. We identify two competitive forces behind LR strategy, which exactly compensate each other in ER networks while yield an interesting crossover phenomenon in SF networks with respect to the scaling exponent *γ*. Our developed analytical framework successfully guides us in choosing more powerful recovery strategy and provides estimation on the desired recovery fraction by using the degree distribution of the original network as the only input.

Note that the analytical and numerical results presented in this work are based on uncorrelated graphs. However, correlations between nodes of similar degree are often found in real-world networks. The tendency to be connected with other nodes with similar degree is referred to as assortative mixing, while disassortative mixing is a bias that high degree nodes tend to attach to low degree nodes^32^,^33^,^34^. Mathematically, the assortativity/disassortativity property can be measured by the Pearson correlation coefficient *ρ* averaged for all pairs of adjacent nodes in the network. In general, *ρ* ∈ [−1, 1] with *ρ* > 0 indicating an assortative network while *ρ* < 0 a disassortative network. In Supplementary Note G, we compare RR and LR strategies on two real social and biological networks. It is revealed that LR seems to be more powerful than RR for healing assortative networks, while the opposite is true for disassortative networks.

This phenomenon can be explained by the dominance of “recovering impetus” in assortative networks in that groups of hub nodes are more likely to be recovered under LR accelerating the recovery process. On the other hand, the disassortative mixing tend to impede the recovery process adding to the “recovering resistance”. We mention that there are other topological properties, such as motif and clustering, that are likely to affect the LR strategy. In addition to numerical study, closed-form results are highly desirable. It is hoped that this work could stimulate further research efforts on the related problems in localized network recovery against damages.

## Methods

Based on the generating function formalism^24^,^26^,^29^,^30^, we are able to solve our theoretical model for the (induced) network composed of failed nodes under RR and LR. We apply our theoretical results to three different types of complex networks: Erdős-Rényi (ER) networks, random regular (REG) networks, and scale-free (SF) networks. An ER network follows a Poisson degree distribution *P*(*k*) = *e*^−*λ*^*λ*^*k*^/*k*! (*k* ≥ 0) with average degree 〈*k*〉 = *λ*. An REG network has a degenerated degree distribution 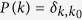, meaning that each node is connected to the same number *k*_0_ of neighbors. A SF network follows a power-law degree distribution 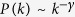 (*k*_min_ ≤ *k* ≤ *k*_max_), where *γ* > 0 is the scaling exponent, *k*_min_ and *k*_max_ mean the minimum and maximum degrees, respectively. For all the simulations, we use networks of size *N* = 10^6^. Moreover, three real-life networks with power-law degree distributions from biological and technological fields are investigated (see Supplementary Note F).

To obtain the *r*_*c*_ curve in the *q*-*r* plane, we first label each node as failed independently with probability 1 − *q* for any given *q* ∈ [0, 1] and inactivate its incident edges. For RR strategy, we begin with *r* = 0 and take a node from the list of failed nodes, change its label to functional with probability *r*, and reactivate each of its incident edges if the other end is connected to a functional node. For LR strategy, we begin with *r* = 0 and take a node from the list of failed nodes in an increasing order according to the distance from a root node, change its label to functional, and reactivate each of its incident edges if the other end is connected to a functional node. After checking the whole list for RR strategy (or respectively, after checking *r* fraction of failed nodes for LR strategy), we then calculate the fraction *P*_∞_ of the giant cluster in the network of failed nodes. Increase *r* and repeat the process until *P*_∞_ < 10^−3^. The *r*_0_ curve is obtained similarly by checking the fraction of the giant cluster in the network of functional nodes.

## Additional Information

**How to cite this article**: Shang, Y. Localized recovery of complex networks against failure. *Sci. Rep.* **6**, 30521; doi: 10.1038/srep30521 (2016).

## Supplementary Material

Supplementary Information

## Figures and Tables

**Table 1:** Three phases for the network composed of occupied (namely, functional and recovered) nodes and three for the “complement network” composed of failed nodes.

Phase	Description
Network of occupied nodes	
I (Non Collapse)	Existence of giant component w/o recovery
II (Recovery)	Existence of giant component w/recovery
III (Collapse)	Non-existence of giant component w/recovery
Network of failed nodes	
A (Non Prevalence)	Non-existence of giant component w/o recovery
B (Restriction)	Non-existence of giant component w/recovery
C (Prevalence)	Existence of giant component w/recovery
