# Supplementary material for: Localized recovery of complex networks against failure
Source: Sci Rep. 2016 Jul 26;6:30521. doi: 10.1038/srep30521 (PMC4960604; doi:10.1038/srep30521)
Supplement: Supplementary Information [file srep30521-s1.pdf]

Localized recovery of complex networks against failure (Supplemental Material)

Yilun Shang
Department of Mathematics, Tongji University, 200092 Shanghai, China

Note A: Derivation for the critical recovery probability r_0(RR)

The random recovery process corresponds to a node percolation with occupation probability $q+r-qr$. Hence, the size distribution of the clusters that can be reached following a randomly selected edge is generated in a self-consistent equation [1]

$$\mathcal{H}_1(x)=(1-q)(1-r)+(q+r-qr)xG_1(\mathcal{H}_1(x)), \tag{S1}$$

where $G_1(x)=G_0'(x)/G_0'(1)$. Similarly, the probability generating function for the size of the cluster to which a randomly selected node belongs is generated by

$$\mathcal{H}_0(x)=(1-q)(1-r)+(q+r-qr)xG_0(\mathcal{H}_1(x)). \tag{S2}$$

Hence, the mean size of small clusters is

$$\mathcal{H}_0'(1)=(q+r-qr)\left(1+\frac{(q+r-qr)G_0'(1)}{1-(q+r-qr)G_1'(1)}\right), \tag{S3}$$

which diverges when $1=(q+r-qr)G_1'(1)$. Note that $q=1/G_1'(1)$ marks the percolation threshold at which a giant cluster first forms without recovery [1]. Eq. (1) follows immediately.

Note B: Derivation for the critical recovery probability r_c(LR) and the fraction of giant component P_∞(LR)

Recall that the original random network contains N nodes and their degrees are generated by $G_0(x)=\sum_{k=0}^{\infty}P(k)x^k$ in the limit of $N\to\infty$. q is the fraction of functional nodes after the initial random failure. We divide the localized recovery process into two regimes: (i) We first recover a fraction r of failed nodes according to LR strategy. Then we assume that

any failed node outside the recovery area are still active (i.e., present). Hence, after the localized recovery process, all nodes and edges in the original network are present; (ii) We remove those failed nodes outside the recovery area. By doing so, we obtain the network of occupied (i.e., functional and recovered) nodes.

Since the initial failure is random, outside the recovery area there are $(1-q)(1-r)N$ failed nodes and $q(1-r)N$ functional nodes, on average, after the LR process. This observation indicates that there is a fraction $(1-r)$ of nodes outside the recovery area. Set $s = 1-r$. We first consider the regime (i). Let $A_s(k)$ be the number of nodes with degree k out of the recovery area. The probability to have a node with degree k out of the recovery area is

$$P_s(k) = \frac{A_s(k)}{sN}. \tag{S4}$$

With one more node being checked, $A_s(k)$ changes as

$$A_{s-1/N}(k) = A_s(k) - \frac{P_s(k)k}{\langle k(s)\rangle}, \tag{S5}$$

where $\langle k(s)\rangle = \sum_k P_s(k)k$. Following [2], in the limit of $N \to \infty$, $(S4)$ and $(S5)$ yield the differential equation

$$-s\frac{dP_s(k)}{ds} = P_s(k) - \frac{P_s(k)k}{\langle k(s)\rangle}. \tag{S6}$$

By direct differentiation, the solution can be expressed as

$$P_s(k) = P(k)\frac{f^k}{G_0(f)}, \tag{S7}$$

and $\langle k(s)\rangle = fG_0'(f)/G_0(f)$, where $f \equiv G_0^{-1}(s)$. We write $G_s(x) = \sum_k P_s(k)x^k = G_0(fx)/G_0(f)$ for the generating function of $P_s(k)$.

Next, instead of considering the regime (ii) by removing failed nodes outside the recovery area, we consider the opposite operation by first (iia) removing the edges connecting the recovery area to outside and then (iib) removing the functional nodes outside the recovery area. Thus, we obtain the "complement network" composed of failed nodes outside the recovery area. $r_c(LR)$ indicates the critical threshold at which a giant component in the complement network first forms.

In the regime (iia), note that the number of edges belonging to the nodes on the outer shell, say, (part of) shell l, of the recovery area minus those connecting inward to shell $l-1$, $L(s)$, can be derived as [2]

$$L(s) = N(G_0'(1)f^2 - G_0'(f)f). \tag{S8}$$

Since loops may exist, the number of edges connecting the recovery area to outside is

$$\tilde{L}(s) = \frac{Ns\langle k(s)\rangle L(s)}{Ns\langle k(s)\rangle + L(s)} = N\left(fG_0'(f) - \frac{G_0'(f)^2}{G_0'(1)}\right). \tag{S9}$$

Due to the randomness of interconnections, the resulting network outside the recovery area can be viewed as the outcome of a bond percolation with occupation probability given by $\tilde{q} = 1 - \tilde{L}(s)/(sN\langle k(s)\rangle) = G_0'(f)/(G_0'(1)f)$. Hence, its probability generating function of nodes' degree distribution, $\tilde{G}_0(x)$, becomes [3, 4]

$$\tilde{G}_0(x) = G_s(1-\tilde{q}+\tilde{q}x) = \frac{1}{G_0(f)}G_0\left(f + \frac{G_0'(f)}{G_0'(1)}(x-1)\right). \qquad (S10).$$

Finally, in the regime (iib), another node percolation is applied with "occupation" probability $1-q$ since the initial failure is random. (Recall that a node is functional with probability q after the initial random failure. Hence, it is removed in regime (iib) with probability q.) Let $\tilde{G}_1(x) = \tilde{G}_0'(x)/\tilde{G}_0'(1)$ be the generating function of the underlying branching process. The size distributions of the clusters that can be reached from a randomly chosen edge, and the clusters that can be traversed by randomly following a starting node are generated, respectively, by [1]

$$\tilde{H}_1(x) = q + (1-q)x\tilde{G}_1(\tilde{H}_1(x)) \qquad (S11)$$

and

$$\tilde{H}_0(x) = q + (1-q)x\tilde{G}_0(\tilde{H}_1(x)). \qquad (S12)$$

The mean size of small clusters is

$$\tilde{H}_0'(1) = (1-q)\left(1 + \frac{(1-q)\tilde{G}_0'(1)}{1-(1-q)\tilde{G}_1'(1)}\right). \qquad (S13)$$

The diverging point of $(S13)$ marks the critical recovery probability $r_c(\mathrm{LR})$, at which a giant component of the complement network emerges. Hence, $r_c = 1 - s_c$ is determined by

$$G_0'(1) = (1-q)G_0''(f), \qquad (S14)$$

where $f \equiv G_0^{-1}(s)$.

The fraction S of the giant component in the complement network of failed nodes outside the recovery area satisfies

$$S(\mathrm{LR}) = 1 - \tilde{H}_0(1) = (1-q)(1-\tilde{G}_0(u)), \qquad (S15)$$

where u satisfies $u = q + (1-q)\tilde{G}_1(u)$. The fraction of the giant component as a fraction of the original network is $P_\infty(\mathrm{LR}) = sS(\mathrm{LR})$.

Note C: Evolution of phase diagrams under RR around the critical point $G_1'(1) = 2$

The point $G_1'(1) = 2$ marks the watershed of the evolution of phase diagrams under RR. Here, we take ER networks as an example, where $G_1'(1) = \lambda$ is just the average degree of

the original network. In Fig. 2(a) we plot the phase diagram for $\lambda = 5$. Fig. S1(a) and Fig. S1(b) below show the phase diagrams for $\lambda = 2$ and $\lambda = 1.6$, respectively. Clearly, when λ decreases from 5 to 2, the three combined phases IB, IC, and IIC contract and vanish simultaneously at the critical point $\lambda = 2$. When λ keeps decreasing, three new combined phases IIA, IIIA, and IIIB emerge simultaneously.

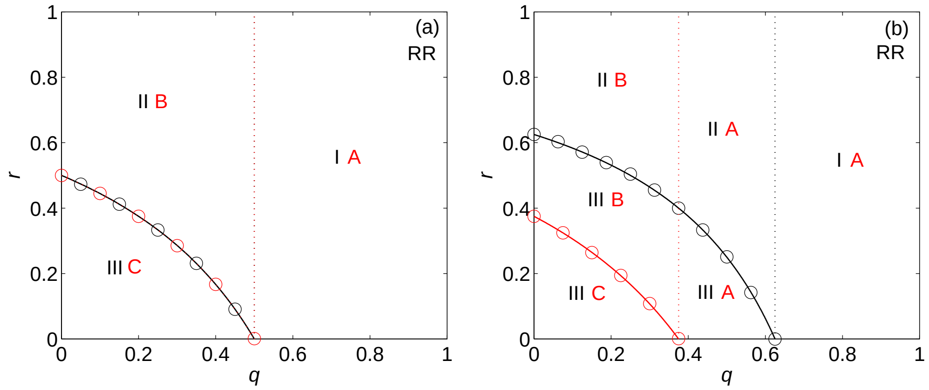

FIG. S1: Phase diagrams in the q-r plane for ER networks under RR for (a) $\lambda = 2$ and (b) $\lambda = 1.6$. The meanings of the combined phases are described in Tab. I. Solid lines are analytical results, from (1) for r_0 (black lines) and (6) for r_c (red lines). Data points (black and red circles) correspond to the simulation results averaged over 30 random graphs with 20 independent realizations for each.

Note D: Proof of $P_\infty(\mathrm{RR}) = P_\infty(\mathrm{LR})$ for all $q, r \in [0,1]$ in ER networks

Let $r_c := r_c(\mathrm{RR}) = r_c(\mathrm{LR})$. For $r \geq r_c$, $P_\infty(\mathrm{RR}) = P_\infty(\mathrm{LR}) = 0$ by definition. Next, we consider the case $r < r_c$. Eqs. (7) and (13) can be recast as

$$\begin{cases} P_\infty(\mathrm{RR}) = (1-q)(1-r)(1-e^{\lambda(w-1)}) \\ w = q + r - qr + (1-q)(1-r)e^{\lambda(w-1)} \end{cases} \tag{$S16$}$$

and

$$\begin{cases} P_\infty(\mathrm{LR}) = (1-q)(1-r)(1-e^{(1-r)\lambda(u-1)}) \\ u = q + (1-q)e^{(1-r)\lambda(u-1)} \end{cases} \tag{$S17$}$$

It is direct to check that ($S16$) is equivalent to ($S17$) by relating w to u following $w = (1-r)(u-1)+1$.

Note E: Phase diagrams for SF networks with $\gamma = 4.5$ and $\langle k \rangle \approx 5$

The (combined) phase diagrams in Fig. S2 are quantitatively similar to those in Fig. 4, in that $r_c(\mathrm{LR}) > r_c(\mathrm{RR})$ for any $q < q_c$, a signature of homogeneous networks.

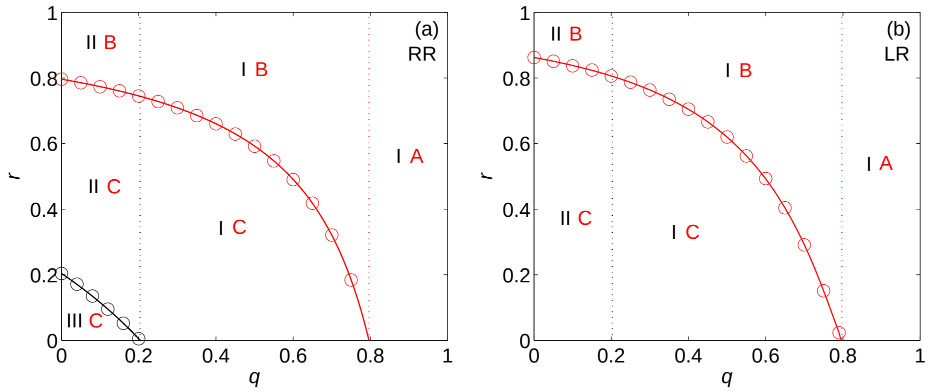

FIG. S2: Phase diagrams in the q-r plane for SF networks with $\gamma = 4.5$, $k_{\min} = 4$, and $\langle k \rangle = 5.01$ under (a) RR and (b) LR strategies. The meanings of the combined phases are described in Tab. I. Solid lines are analytical results, from (1) for r_0 (black line) and (4), (10) for r_c (red lines). Data points (black and red circles) correspond to the simulation results averaged over 30 random graphs with 20 independent realizations for each.

			analytical results				simulated results			
	q	γ	γ_c	γ_∞	$\hat{r}_c$	$\hat{P}_\infty$	r_c(RR)	P_∞(RR)	r_c(LR)	P_∞(LR)
Internet	0.3	2.1	2.9	3.4	0.48	0.25	0.69	0.31	0.43	0.21
Circuit	0.6	3	2.7	2.9	0.64	0.33	0.51	0.25	0.70	0.36
Protein	0.1	2.4	3.0	3.3	0.56	0.38	0.62	0.44	0.50	0.27

TABLE S1: Results for three empirical scale-free networks under RR and LR. The recovery fraction r is set to 0.1 for all three networks when calculating γ_∞, $\hat{P}_\infty$, P_∞(RR) and P_∞(LR).

Note F: Predicting robustness of real networks under RR and LR

Three real-world scale-free networks are studied here. The first is Internet [5], which maps the Internet topology at the autonomous system level. Each node in Internet represents an administrative domain and each edge indicates the existences of a Border Gateway Protocol peer connection. It has $N = 11174$ nodes, $\langle k \rangle = 4.18$, and $\gamma = 2.1$. The second is Circuit [6], which is the network of electronic circuits with nodes being electronic components and edges being wires. It has $N = 24097$ nodes, $\langle k \rangle = 4.34$, and $\gamma = 3$. The third is Protein [7], which is the protein interaction network with nodes being proteins and edges biological interactions. It has $N = 2115$ nodes, $\langle k \rangle = 2.12$, and $\gamma = 2.4$.

At a given level of initial failure q in the network, we determine the critical exponent γ_c by requiring $r_c(\text{RR}) = r_c(\text{LR})$ at the value of q. This critical recovery fraction is denoted by $\hat{r}_c$. Analogously, we write $\hat{P}_\infty := P_\infty(\text{RR}) = P_\infty(\text{LR})$ on identifying the critical exponent

γ_∞ so that $P_\infty(\mathrm{RR}) = P_\infty(\mathrm{LR})$ holds at given q and r. The results are summarized in Table S1.

For Internet, we find that $\gamma_c = 2.9 > \gamma = 2.1$ in Table S1. This implies that the "recovering impetus" is dominant when $q = 0.3$, and LR is the better strategy. This conclusion is supported by the numerical result $r_c(\mathrm{RR}) = 0.69 > r_c(\mathrm{LR}) = 0.43$. The theoretical critical recovery fraction $\hat{r}_c = 0.48$ represents a good approximation (in fact, an upper bound) for $r_c(\mathrm{LR})$. Similar analysis can be applied to γ_∞, $\hat{P}_\infty$, and the networks Circuit and Protein. In all the situations considered, our theoretical predictions are found to be supported very well by the numerical simulations, and thus can guide us in choosing appropriate recovery strategy and provide estimation on the desired recovery fraction by using the degree distribution of the original network as the only input.

Note G: RR and LR on correlated networks

We compare RR and LR on two real-life correlated networks. The first is a metabolic network Reactome, which has $N = 5973$ nodes [8]. Its Pearson correlation coefficient is shown to be $\rho = 0.24$ and hence is an assortative network. The second is a social network describing Facebook user-user relationship called Facebook [9]. It has $N = 2888$ nodes and Pearson correlation coefficient $\rho = -0.67$. Therefore, Facebook is a disassortative network.

Fig. S3 shows that $P_\infty(\mathrm{RR}) > P_\infty(\mathrm{LR})$ for Reactome. The difference between $P_\infty(\mathrm{RR})$ and $P_\infty(\mathrm{LR})$ is prominent. For example, under a relatively sever random error with $q = 0.2$, the giant cluster in the network of failed nodes in Reactome has more than 30% nodes when half of the failed nodes are recovered by RR, but has only about 2% nodes when using LR strategy. Evidently, LR is much more powerful than RR for healing Reactome. On the contrary, we observe that $P_\infty(\mathrm{LR}) > P_\infty(\mathrm{RR})$ for Facebook, which suggests that disassortative mixing may hinder the LR process. This agrees with our intuition that low degree nodes linked to a recovered hub would reduce the recovery progression under LR since low degree nodes have limited contribution to the giant component of occupied nodes.

[1] D. S. Callaway, M. E. J. Newman, S. H. Strogatz, and D. J. Watts, Phys. Rev. Lett. **85**, 5468 (2000).

[2] J. Shao, S. V. Buldyrev, L. A. Braunstein, S. Havlin, and H. E. Stanley, Phys. Rev. E **80**, 036105 (2009).

[3] S. Shao, X. Huang, H. E. Stanley, and S. Havlin, New J. Phys. **17**, 023049 (2015).

[4] M. E. J. Newman, Phys. Rev. E **66**, 016128 (2002).

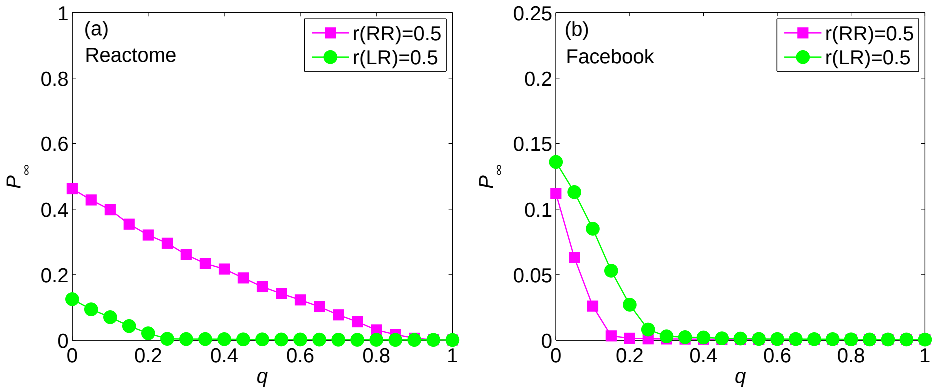

FIG. S3: Relative sizes of giant component in the network of failed nodes, P_∞, as a function of q for (a) Reactome network and (b) Facebook network with $r(\mathrm{RR}) = 0.5$ (squares) and $r(\mathrm{LR}) = 0.5$ (circles). The simulation results are averaged over 20 independent realizations.

[5] A. Vázquez, R. Pastor-Satorras, and A. Vespignani, Phys. Rev. E **65**, 066130 (2002).

[6] R. F. i Cancho, C. Janssen, and R. V. Solé, Phys. Rev. E **64**, 046119 (2001).

[7] H. Jeong, S. Mason, A.-L. Barabási, and Z. N. Oltvai, Nature **411**, 41 (2001).

[8] G. Joshi-Tope, M. Gillespie, I. Vastrik, P. D'Eustachio, E. Schmidt, B. de Bono, B. Jassal, G. R. Gopinath, G. R. Wu, L. Matthews, et al., Nucl. Acids. Res. **33**, D428 (2005).

[9] J. Leskovec and J. J. McAuley, in *Advances in Neural Information Processing Systems* (2012), pp. 548–556.
